# Editorial: Astrobiology at the Interface: Interactions Between Biospheres, Geospheres, Hydrospheres and Atmospheres Under Planetary Conditions

**DOI:** 10.3389/fmicb.2021.629961

**Published:** 2021-02-12

**Authors:** Tetyana Milojevic, Adrienne Kish, Akihiko Yamagishi

**Affiliations:** ^1^Space Biochemistry Group, Department of Biophysical Chemistry, University of Vienna, Vienna, Austria; ^2^Muséum National d'Histoire Naturelle, Paris, France; ^3^Department of Applied Life Sciences, School of Life Sciences, Tokyo University of Pharmacy and Life Sciences, Hachioji, Japan

**Keywords:** astrobiology, outer space, extremophiles, ISS, Universe, Mars, space simulating facilities, biosignatures

Astrobiology is a young, rapidly developing branch of science that seeks to address the question of whether life exists, or has existed, elsewhere in the Universe. It is by nature an interdisciplinary field that explores the origins of life, the conditions and processes that support or challenge life, the influence of different environmental conditions on the preservation and detection of biosignatures of past and present life, and the spread of life across the Universe.

As highly interdisciplinary field, astrobiology requires an integrative approach to link the efforts of microbiologists exploring the origins, evolution, and limits of life to the work of geologists exploring both planetary conditions (past and present) and preservation of biosignatures in the geological record. In the search for potential life on other planetary bodies, efforts are being made to combine exoplanet discovery, the study of asteroids and comets, ground-based analyses of recovered meteorites, microbial space exposure experiments with analog planetary-scale biosignature detection. Being a few steps in front of retrieving and returning the first samples from the surface of Mars, we have already gained extensive knowledge from the field, laboratory, and space exposure experiments (McKay and Stoker, 1989; Grotzinger and Milliken, 2012; Westall et al., 2015; Hays et al., 2017). This should enable a comprehensive characterization of the first Mars returned samples in terms of potential exobiology.

Outer space, along with ground-based simulating facilities, provides a research tool for studying life in the Universe. Multiple microbial exposure experiments have been successfully performed on board and outside of the International Space Station under the environmental conditions of low Earth orbit (Cockell et al., 2011; Nicholson et al., 2012; Vaishampayan et al., 2012; Kawaguchi et al., 2020; Ott et al., 2020) or mimicking planetary constraints (galactic cosmic and solar UV radiation, temperature fluctuations, microgravity, freezing, desiccation, and extreme vacuum) (Nicholson et al., 2012; Billi et al., 2019; de Vera et al., 2019; Panitz et al., 2019). Exposure experiments at ground-based simulating facilities have enabled the investigation of the effects of space-related parameters on microbial survival and adaptation capacities (Mastroleo et al., 2013; Ott et al., 2019a,b; Beblo-Vranesevic et al., 2020).

Revealing unknown boundaries for prokaryotic life under multiple extremes is a prerequisite to understanding the extent of biology on Earth, and to discover its possible wider presence in the Universe. Understanding freezing tolerance and survival limits of thermophiles in permanently cold habitats is important for studies of microbial transfer through space and between celestial bodies. The peculiar presence of thermophiles in permanently cold marine and terrestrial habitats, including Arctic marine sediments (Hubert et al., 2009; Hanson et al., 2019), cold seawaters (Mora et al., 2014; Wirth, 2017), Antarctic accretion ice (Bulat et al., 2004; Lavire et al., 2006), permafrost (Gilichinsky et al., 2007; Demidov and Gilichinsky, 2009), and laboratory investigations of thermophiles under low temperatures (Marchant et al., 2008; Milojevic et al., 2020) has been known for many years. However, our understanding of the molecular and physiological mechanisms of thermophilic adaptation to low temperatures is still poor.

The papers in this Research Topic cover a range of microbiological and biochemical research in extreme environments, from cold marine sediments with permanent low temperatures to hot springs in Yellowstone National Park, and to drilling samples from millions-year-old oceanic crust with deep fractured rock ecosystems. These papers provide a snapshot of current research activities in the field of astrobiology that have evolved at the boundaries of biosphere, geosphere, hydrosphere and atmosphere. In this research collection we present one review and 8 original research papers exploring the interactions between the biological, geological, hydrological, and atmospheric elements in the Universe. These works seek to address the impact of planetary conditions on the evolution of microorganisms, molecular mechanisms driving the limits of life under different physiochemical regimes, and traces of life that can be detected in the physiochemical conditions of Earth and beyond.

Several papers in this collection are addressing the survival limits of extremophiles exposed to harsh space-related parameters and drastic planetary constraints. Mosca et al. studied the desert cyanobacterium *Chroococcidiopsis* exposed to a Mars-like UV flux and long-termed desiccation. Their study reshaped the boundaries of *Chroococcidiopsis* desiccation and UV tolerance and revealed several molecular determinants of DNA damage and repair response of *Chroococcidiopsis*.

A review by Milojevic and Weckwerth presents further detailed analysis of molecular mechanisms behind microbial survival and adaptation to an outer space environment. This review is focused on molecular strategies revealed with the help of the global and integrative –omics approaches of systems biology that have been recently used to study microorganisms exposed to real and simulated space conditions. Popall et al. investigated the influence of UV-C radiation on cultures dominated by the cyanobacterium *Geitlerinema* sp., which was obtained from a laboratory-maintained stromatolite originating from a Precambrian analog lagoonal system Lagoa Vermelha, Brazil. The *Geitlerinema* sp. was able to withstand long-term exposure to UV-C radiation, building and maintaining filamentous mats after harmful irradiation.

The other papers in this Research Topic issue illustrate co-evolution of biosphere, geosphere, and hydrosphere, and consider biosignatures of life formed at their interfaces. Boyer et al. analyzed the influence of redox geochemistry on lipid composition of microbial communities inhabiting hot springs in Yellowstone National Park. This study proposed chemical signatures in lipid biomarkers that could potentially represent a fingerprint of the geochemical paleoredox conditions on microorganisms inhabiting these paleoenvironments. Sueoka et al. investigated a basaltic rock core sample of 104-million-year-old oceanic crust and proposed that deep saponite-bearing fractures could harbor extant life and/or host the remnants of bygone life on Mars. Hamilton-Brehm et al. described a novel anaerobic and thermophilic bacterium *Thermoanaerosceptrum fracticalcis* isolated from deep fractured rock ecosystems of the US Great Basin and provided insights into metabolic strategies of the deep subsurface biosphere. Glamoclija et al. studied subsurface microbial ecology of hypersaline playa Lake Lucero at the White Sands National Monument in New Mexico and proposed this region as a terrestrial analog for future astrobiological explorations. The limits of microbial life in saline and cold environments with potential implications for habitability of Martian cryobrines were a focus of investigations by Waajen et al. Their laboratory investigations showed that salt concentration, anion parameters and the water activity are crucial factors in the survival of the cryo- and halotolerant bacterial strain *Planococcus halocryophilus* in concentrated brines. Thus, anion brine composition, the salt concentration and water activity were proposed as a set of environmental parameters to be considered when investigating potential habitability of Martian cryobrines. Microbial life under extremely low temperatures was also investigated by Cramm et al., who studied the influence of freezing temperatures on the survival of thermophilic endospore-forming bacteria. Their laboratory study showed that thermospores remain viable after freezing at temperatures as low as −80°C, making them suitable for microbial viability investigations in Martian surface soil with temperature fluctuations between 20 and −76°C.

In summary, this Research Topic includes both novel research strategies and methodologies that should yield promising results in the future, expanding our current view of life-search across the Universe.

## Author Contributions

The authors are co-editors which organized this Research Topic, substantially, directly, and intellectually contributed to the work on this Research Topic and the manuscript, and approved it for publication. All authors contributed to the article and approved the submitted version.

## Conflict of Interest

The authors declare that the research was conducted in the absence of any commercial or financial relationships that could be construed as a potential conflict of interest.
